# The Individual Placement and Support (IPS) in Pain Trial: A Randomized Controlled Trial of IPS for Patients with Chronic Pain Conditions

**DOI:** 10.1093/pm/pnac032

**Published:** 2022-03-02

**Authors:** Vigdis Sveinsdottir, Henrik Børsting Jacobsen, Tone Marte Ljosaa, Lene Therese Bergerud Linnemørken, Thomas Knutzen, Reza Ghiasvand, Silje Endresen Reme

**Affiliations:** Department of Pain Management and Research, Oslo University Hospital, Oslo, Norway; NORCE Norwegian Research Centre, Bergen, Norway; Department of Pain Management and Research, Oslo University Hospital, Oslo, Norway; The Mind Body Lab, Department of Psychology, University of Oslo, Oslo, Norway; Department of Pain Management and Research, Oslo University Hospital, Oslo, Norway; Department of Pain Management and Research, Oslo University Hospital, Oslo, Norway; Department of Research and Development, Division of Emergencies and Critical Care, Oslo University Hospital, Oslo, Norway; IPS Norge AS, Oslo, Norway; Oslo Centre for Biostatistics and Epidemiology, Oslo University Hospital, Oslo, Norway; Department of Research, Cancer Registry of Norway, Oslo, Norway; Department of Pain Management and Research, Oslo University Hospital, Oslo, Norway; The Mind Body Lab, Department of Psychology, University of Oslo, Oslo, Norway

**Keywords:** Chronic Pain, Individual Placement and Support, Integrated Care, Randomized Controlled Trial, Work Disability

## Abstract

**Objectives:**

Although complex pain conditions require an interdisciplinary approach, employment services are rarely provided in pain centers. Individual Placement and Support (IPS) is an effective approach to increase work participation among patients with severe mental illness, and recent evidence suggests that this method can be successfully repurposed for new target groups. We aimed to investigate the effectiveness of IPS integrated with interdisciplinary treatment as usual (TAU) for patients with chronic pain in a tertiary pain center.

**Methods:**

A randomized controlled trial comparing IPS integrated with TAU (n = 38) with TAU alone (n = 20) was conducted. Participants were patients with chronic pain who were 18–65 years of age and currently on long-term sick leave or disability benefits or unemployed. The primary outcome was employment within 12 months after enrollment, with additional long-term follow-up after 24 months. Secondary outcomes included health and quality of life, measured at baseline, 6 months, and 12 months.

**Results:**

During 12-month follow-up, 52.8% in the IPS group and 38.9% in the TAU group had attained employment. The difference increased during 24-month follow-up but did not reach statistical significance. Findings on secondary outcomes were generally nonsignificant.

**Conclusions:**

The IPS in Pain trial is the first study to evaluate the effect of IPS for patients with chronic pain conditions. It shows that IPS can be integrated into the daily practice of interdisciplinary pain treatment, with employment rates exceeding 50% in 1 year and a clear trend in favor of the IPS group. Results did not, however, reach significance. Larger randomized controlled trials are needed to draw clear conclusions about effectiveness.

## Introduction

Chronic pain represents a large and growing international health problem [1], accounting for substantial social expenditures related to treatment and benefit recipiency. In Norway, pain is the leading cause of nonfatal loss of health and reduced work life participation: Up to 50% of disability benefit cases are attributed to chronic pain, which is also the main diagnosis in 35–45% of doctor-certified sick leaves [2].

People with chronic pain can face many obstacles in finding employment or returning to work after a period of absence [3, 4]. The importance of employment for health and well-being has been extensively documented [5–10], and being excluded from working life deprives the individual of important employment-related functions, such as the organization of daily activities, social contact, and social identity [11]. Complex pain conditions require a multidisciplinary approach [12], which may include analgesics to alleviate symptoms; physical exercise to improve, for example, muscle strength and balance; and cognitive behavioral techniques to enhance coping [13]. Such approaches have been implemented in pain centers for years, but, despite the significant role of employment in health and well-being, evidence-based employment services are rarely provided in this context.

Individual Placement and Support (IPS) is an evidence-based intervention originally designed to help people with severe mental illness (SMI), e.g., schizophrenia, gain and keep employment. IPS has been shown to be effective in the original target group across 28 randomized controlled trials (RCTs), with mean employment rates of 55%, as compared with 25% in control conditions [14]. Recent studies also have suggested that the method can be successfully applied to new target groups [15]. The effect of IPS has not been investigated in patients with chronic pain, apart from a recent pilot study indicating its suitability. In that study, IPS services were offered as an integrated part of interdisciplinary pain treatment for eight patients who had been out of working life for 2–16 years [16]. Results suggested that integration of IPS was feasible and participants were generally satisfied with the intervention. During a 12-month follow-up period, three of eight participants gained *competitive employment* (i.e., ordinary paid work in the competitive labor market), while one participant dropped out of the study. Although findings from the pilot study were encouraging, there was a need to investigate the effectiveness of IPS for patients with chronic pain in a larger RCT.

The present trial aimed to investigate whether IPS as an integrated part of the interdisciplinary treatment as usual (TAU) for patients with chronic pain in a tertiary pain center resulted in a higher rate of competitive employment (research question 1), higher job productivity (research question 2), or more improvement in health and quality of life (QoL) (research question 3) than did TAU alone.

## Methods

### Trial Design

The IPS in Pain trial was an RCT comparing pain treatment with integrated IPS to TAU. The project was submitted to the Norwegian Regional Ethical Committee, and from there it was referred to the Data Protection Officer at Oslo University Hospital, who approved the study (project number: 2015/14224). The trial was registered at ClinicalTrials.gov (NCT02697656), and a detailed study protocol was published [17].

### Participants and Recruitment

Eligible participants were patients referred to the pain center at the Department of Pain Management and Research at Oslo University Hospital in Norway during the recruitment period (November 2015 through December 2017). The inclusion criteria were: eligible for interdisciplinary treatment, not employed (i.e., on long-term sick leave, receiving benefits because of impaired work capability or disability, or unemployed), expressed desire to work, age 18–65 years, living in Oslo, and ability to answer questionnaires in Norwegian.

All patients referred to the center were informed of the study and invited to participate. Eligible patients who wished to participate received additional information about the procedures and purposes of the study and their right to withdraw at any time, and they provided informed consent to participate.

### Interventions

Participants were randomly assigned to either an intervention group (IPS) or a control group (TAU). The randomization process was described in the study protocol [17].

The intervention group received job support according to the IPS model from an employment specialist, integrated with the usual interdisciplinary pain treatment provided at the pain center. Two employment specialists were hired at the center during the trial period, specifically in relation to the project. In addition to regular ad hoc meetings, the employment specialists and pain management team had meetings during which they discussed all participants. The employment specialists delivered job support to the participants according to the supported employment fidelity review manual [18], adhering to the eight principles of IPS: 1) eligibility based on the individual’s own choice, 2) focus on competitive employment (meaning jobs in the competitive labor market, that pay regular wages, and that anyone can apply for regardless of disability status), 3) integration of mental health and employment services, 4) attention to individual preferences, 5) work incentives planning, 6) rapid job search, 7) systematic job development, and 8) long-term individualized support. In line with the IPS methodology [18], support from the employment specialist could take place in various community settings, at the clinic, or at the workplace (if/when participants were employed).

Because IPS was originally developed for people with SMI, some adaptations were necessary to serve the population in the present study. The adaptations were made in line with recommendations in the fidelity review manual [18]. With regard to the principle of integration of services, the employment services were integrated with the interdisciplinary pain treatment and not mental health treatment per se, although the treatment team could involve a psychologist. The principle of work incentives planning was handled by hiring a coordinator from the Norwegian Labor and Welfare Administration (NAV) during the trial period, to supplement the services provided by the employment specialists. The NAV coordinator was located at the pain center with access to the data systems of all local NAV offices and gave general advice on benefits and work incentives. The position had the function of a case manager and was the point of contact between NAV and both participants and treatment providers.

IPS services were provided for up to 2 years for the first included participants and for up to 1 year for participants included later in the project period. Fidelity to the IPS model was assessed at four time points during 2016–2017. The fidelity assessments were conducted by an independent and experienced evaluator using the established Norwegian translation of the 25-item IPS fidelity scale and are described in more detail in the study protocol [17]. Scores ranged from 71 (below the accepted threshold of IPS) in the first two assessments to 86 and 89 (fair fidelity) in the two later assessments, with a mean score of 79, signifying fair fidelity to the IPS model.

The control group received TAU, consisting of interdisciplinary pain treatment provided at the center by physicians (anesthesiologist, gynecologist, neurologist, or specialist in physical medicine and rehabilitation), psychologists, physiotherapists, and nurses. In TAU, at least two different professions followed the patients for up to 1 year, with a frequency of every other week to once a month. Participants in TAU also received a resource manual with information about services and resources for people in unemployment or work disability, self-help advice, and information about pain management. Furthermore, participants who were eligible for vocational rehabilitation provided by the NAV were advised to contact their local NAV office to receive employment services.

### Outcomes

#### Competitive Employment

The primary outcome was the rate of competitive employment during follow-up, with 12 months after enrollment as the primary follow-up period. In line with previous trials of IPS [19], this outcome was defined as the percentage of participants in each group who obtained ordinary paid employment in the competitive labor market during the follow-up period, with a threshold of a least 1 day of work. Dichotomous variables indicating any competitive employment vs no competitive employment were created. Long-term follow-up data on the primary outcome were also collected at 24 months.

Additional standardized indicators of success in employment were the percentage of participants working ≥20 hours in 1 week (which corresponds to at least half-time employment), total hours worked or wages, and the number of weeks employed during the 12-month follow-up [19]. Data on wages and employment duration were incomplete and are not reported. Employment specialists also recorded the types of jobs attained among participants in the IPS group.

#### Health-Related Outcomes

Secondary health-related outcomes were measured through the use of various questionnaires at baseline, 6-month follow-up, and 12-month follow-up.

*Health-related* *QoL*. The EuroQol Visual Analogue Scale (EQ-VAS) was used to measure self-reported health-related QoL. The EQ-VAS is a vertical scale ranging from 0 (worst imaginable health state) to 100 (best imaginable health state) [20]. Although there is a need for more high-quality evidence to assess its reliability, the measure has shown acceptable validity as a generic health-related measure of QoL [21].

*Pain-related disability* *.* A modified version of the Oswestry Disability Index (ODI) was used to measure self-reported disability related to pain. The modification consisted of deleting the word “back” in order to measure pain more generally. The ODI has proved to be a valid and reliable measure of disability [22, 23] and contains 10 items concerning the effect of pain on different activities in daily life. Each item was scored on a scale from 0 to 5, where higher values represent more disability.

*Psychological distress.* The Hopkins Symptom Checklist-25 (HSCL-25) was used to measure self-reported psychological distress [24]. The scale consists of 25 items, includes subscales of anxiety and depression, and has been shown to have satisfactory validity and reliability [25]. Each item was scored on a scale from 1 to 4, where higher values represent more severe symptoms, with a mean cutoff score of ≥1.75 to indicate psychological distress [25].

*Pain intensity*. Numeric Rating Scales (NRS) from the Brief Pain Inventory (BPI) were used to measure the participants’ pain intensity. The BPI has demonstrated reliability and validity as a tool for clinical pain assessment and for assessment of the effectiveness of pain treatment [26]. Participants were asked to rate their current, worst, average, and least pain during the prior week, on four scales from 0 (no pain) to 10 (worst imaginable pain). These scales replaced the variables of pain intensity and bothersomeness as described in the protocol article, which were not available because of an error in the data collection.

### Data Collection and Management

Baseline data were collected at the pain center via an electronic Lenovo tablet with secure software or through the use of paper questionnaires that were subsequently entered electronically by the trial coordinator. At the 6- and 12-month follow-ups, participants chose whether to complete the questionnaires at the pain center or at home. To increase response rates, nonrespondents were contacted by phone and were also asked verbally about the primary outcome. Logbooks from the employment specialists were used to obtain missing information on the primary outcome for participants in the IPS group.

After 24 months, long-term follow-up data on the primary outcome were collected through a brief phone interview with the trial coordinator or a research assistant.

For more information about data collection, procedures, and management, see the study protocol [17].

### Sample Size

Sample size calculations were based on previous IPS trials showing competitive employment rates of 61% for IPS groups and 23% for control groups [27], with a 5% significance level and a power of 90% [17]. This would require n = 31 in each group. Considering that IPS had not been previously investigated for a chronic pain population and that any effects might be more moderate than those found in earlier studies, we aimed to recruit 80 participants in total.

### Randomization

After baseline assessment, participants were randomly assigned to one of the two groups. Randomization was conducted by the research team using the app “RandomizeIt” with a 1:1 randomization ratio. During the first months of recruitment, a 2:1 ratio was applied to ensure a sufficient caseload for the IPS employment specialists. Participants were informed of the randomization outcome by the principal investigator or the trial coordinator.

### Statistical Analyses

Descriptive statistics were used to present baseline demographic and clinical characteristics for participants in each group. Baseline differences between groups were analyzed with Pearson chi-squared tests and independent-samples *t* tests.

To test research question 1, chi-squared tests were used to compare the proportions of participants in each group who had been employed since baseline at the 6-, 12-, and 24-month follow-up points. In these tests, odds ratios (ORs) and 95% confidence intervals (CIs) were also calculated. A graph providing a visual representation of the employment rates in each group at each follow-up point was created.

To test research question 2, the total numbers of hours worked during 12-month follow-up among participants in each group were compared with *t* tests. Chi-squared tests, ORs, and 95% CIs were used to compare the proportions of participants in each group who had ever worked ≥20 hours per week during 12-month follow-up. In cases in which one cell had an expected cell count less than 5, exact *P* values (Fisher’s exact test significance) were used.

To test research question 3, *t* tests were used to analyze differences between the groups on health-related outcomes at the 6- and 12-month follow-up points. However, because of lower response rates on these outcomes at follow-up, as well as the multiple observations for participants at baseline, 6 months, and 12 months, additional analyses with mixed-effects regression models were conducted to adjust for baseline and missing data. In these analyses, maximum likelihood estimation will robustly adjust for missing observations. Using this approach accounts for complex structures of missing data [28].

The statistician and a researcher who carried out quality control of the data analyses were blinded for intervention assignment.

Analyses were performed in IBM SPSS Statistics version 25 (Armonk, NY, USA: IBM Corp) and Stata version 16 (College Station, TX, USA: StataCorp LLC) and followed the intention-to-treat principle according to the randomized groups. The significance level was α = 0.05.

### Ancillary Analysis: Historical Control Group

Because of practical and implementation issues, we did not reach our target number of participants in the trial. The relatively low response rate at follow-up further contributed to reduce the statistical power. We thus decided to add an additional comparison with a historical control group, which was based on data on the general clinical population from the Oslo University Hospital Pain Registry [29]. This registry includes information collected from all patients at the pain center at the time of their first scheduled consultation. The general clinical population had demographic and clinical characteristics similar to those of the trial participants in the IPS group [29]. The historical control group was selected by work status at baseline (receiving benefits because of impaired work capability or disability) and by the presence of a response to a question about their current employment status after 12 months at the pain center (n = 315). Those who reported being employed at 12-month follow-up were asked to indicate full-time or part-time work percentage. The employment rate among patients in the historical control group was compared with that in the IPS group with Pearson’s chi-squared test, OR, and 95% CI.

The analysis including the historical control group was a secondary and ancillary analysis. The historical controls were not included in the main analysis.

### Ethics Considerations

The project was submitted to the Norwegian Regional Ethical Committee, from which it was referred to and approved by the Data Protection Officer at Oslo University Hospital (project #2015/14224). The principles of the Helsinki Declaration were followed, and all participants provided written informed consent. Confidentiality was guaranteed for all participants, and personal information was securely stored in a locked and fireproof safe.

## Results

### Participant Flow and Characteristics

**Figure 1.**
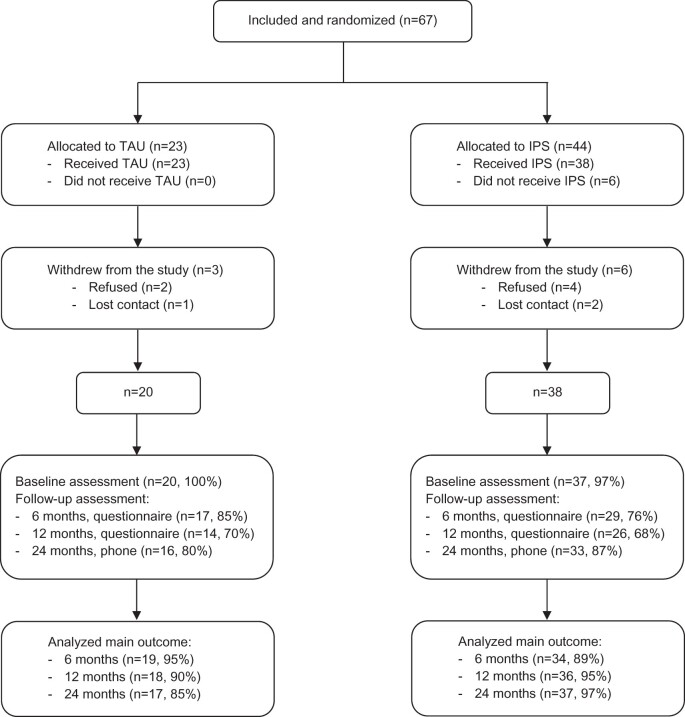
Participant flow.

A total of 67 participants were included and randomized, after which nine withdrew, leaving 58 participants in the final sample (IPS group: n = 38, TAU group: n = 20). See Figure 1 for a participant flowchart.

Baseline demographic and clinical characteristics for each group are presented in Table 1. The groups did not differ significantly in any characteristics at baseline.

**Table 1.** Baseline characteristics for the IPS and TAU groups

	IPS (n = 38)		TAU (n = 20)		
Characteristic	n (%)*	Mean (SD)	n (%)*	Mean (SD)	*P*
Female	29 (76.3)		14 (70)		0.602
Age, years		42.63 (12.20)		42.85 (10.18)	0.946
Higher education	14 (40)		12 (60)		0.153
Married / living with partner	11 (32.3)		9 (47.4)		0.279
Has children	19 (55.9)		12 (63.2)		0.606
Benefit recipiency	32 (84.2)		16 (80)		
Work Assessment Allowance	21 (65.6)		10 (62.5)		
Disability benefits	5 (15.6)		3 (18.8)		
Other	6 (18.7)		3 (18.8)		
Been out of working life >2 years	21 (72.4)		13 (81.3)		
Pain-related disability (0–100)		38.61 (16.15)		45.68 (16.34)	0.126
Health-related QoL (0–100)		44.12 (16.36)		44.53 (21.68)	0.939
Psychological distress (1–4)		2.45 (0.57)		2.30 (0.51)	0.357
Above cutoff	32 (97)		16 (88.9)		0.282^†^
Anxiety (1–4)		2.20 (0.60)		2.03 (0.50)	0.312
Depression (1–4)		2.61 (0.61)		2.47 (0.57)	0.446
Pain intensity in prior week (0–10)					
Current		5.26 (2.37)		5.65 (2.16)	0.544
Worst		7.50 (1.78)		7.80 (1.44)	0.525
Average		5.42 (1.80)		6 (1.56)	0.242
Least		3.44 (2.25)		4.15 (2.43)	0.282

SD= standard deviation.

* Percentages are reported as valid percent based on the total number of respondents to each outcome at baseline.

† Two cells had an expected cell count less than 5. Exact *P* value (Fisher’s exact test significance) was used.

### Primary Outcome: Competitive Employment

Participants in the IPS group reported a higher rate of competitive employment at all follow-up points, but the differences were not statistically significant (Table 2).

**Table 2.** Proportion of participants in each group employed up to each follow-up point and comparison of the groups

Follow-Up Point	IPS n (%)*	TAU n (%)*	*P*	OR (95% CI)
6 Months	11 (32.4)	6 (31.6)	0.954	1.04 (0.31–3.46)
12 Months	19 (52.8)	7 (38.9)	0.336	1.76 (0.56–5.56)
24 Months	22 (59.5)	7 (41.2)	0.211	2.10 (0.65–6.74)

* Percentages are reported as valid percent based on the total number of respondents at each time point.

**Figure 2.**
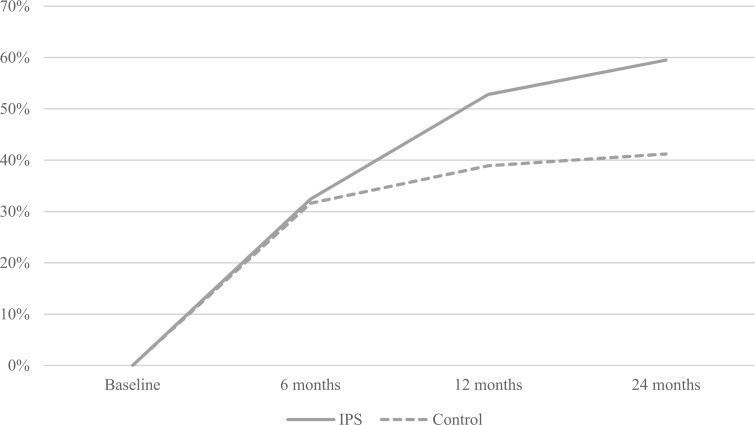
Employment rates in each group during 6-, 12-, and 24-month follow-up.

The graph in Figure 2 illustrates how both groups showed increasing employment rates throughout the follow-up period, but this trend was more pronounced among participants in the IPS group.

### Additional Indicators of Success in Employment

Mean numbers of hours worked were 216.53 in the IPS group (standard deviation = 447.25, n = 35, range: 0–1800) and 122.94 in the control group (standard deviation = 255.20, n = 17, range: 0–900), but the difference was nonsignificant (*P* = 0.428). The proportions of participants working ≥20 hours in a week were 17.65% (n = 6) in the IPS group and 11.76% (n = 2) in the control group, but this difference was also nonsignificant (Fisher’s exact *P* = 0.703, OR = 1.61, 95% CI = 0.29–8.96).

The types of jobs attained among IPS participants were mainly within health and social services (23%), child care and school (13%), assistants in various businesses (13%), and shop and retail (10%). Remaining jobs were scattered across diverse sectors in the labor market.

### Secondary Health-Related Outcomes

In the unadjusted analyses, there were no statistically significant group differences in secondary outcomes at the 6- or 12-month follow-up points, with the exception of worst pain during the prior week (Table 3).

**Table 3.** Unadjusted secondary outcomes and comparison of the groups at each follow-up point

	6 Months					12 Months				
	IPS		TAU			IPS		TAU		
Outcome	n	Mean (SD)	n	Mean (SD)	*P*	n	Mean (SD)	n	Mean (SD)	*P*
Pain-related disability (0–100)	28	36.09 (9.71)	17	40.96 (17.27)	0.298	25	39.18 (9.23)	14	37.79 (17.44)	0.785
Health-related QoL (0–100)	29	47.41 (16.16)	17	42.76 (21.08)	0.405	26	39.42 (17.05)	14	42.07 (17.87)	0.648
Psychological distress (1–4)	28	2.33 (0.61)	16	2.21 (0.71)	0.557	25	2.43 (0.64)	14	2.13 (0.62)	0.163
Anxiety	28	2.14 (0.64)	16	2.03 (0.63)	0.560	25	2.28 (0.66)	14	1.90 (0.45)	0.060
Depression	28	2.46 (0.68)	16	2.34 (0.81)	0.590	25	2.53 (0.68)	14	2.29 (0.75)	0.307
Pain intensity in prior week (0–10)										
Current	28	5.25 (2.15)	16	5.69 (3.05)	0.617	25	5.28 (1.81)	14	5.43 (2.98)	0.867
Worst	28	7.64 (1.66)	16	7.75 (2.11)	0.853	25	8.28 (1.37)	14	6.86 (1.99)	***0.012***
Average	28	5.43 (1.57)	16	5.94 (2.46)	0.407	25	6.16 (1.65)	14	5.21 (2.22)	0.139
Least	28	3.68 (1.87)	16	4.13 (3.18)	0.613	25	4.00 (2.29)	14	3.21 (2.04)	0.293

SD = standard deviation.

The analyses with adjustment for baseline and missing data showed that differences between the groups remained nonsignificant. In these analyses, worst pain during the prior week was also nonsignificant (Table 4).

**Table 4.** Secondary outcomes adjusted for baseline and missing data

Outcome	Estimate*	*SE*	*F* Score	*P* Value
Pain-related disability (0–100)	–3.53	3.82	–0.92	0.356
Time	–1.13	1.07	–1.05	0.292
Interaction group×time	4.05	2.17	1.86	0.062
Intercept	42.78	3.30	12.95	0
Health-related QoL (0–100)	0.46	4.18	0.11	0.913
Time	–1.83	1.69	–1.08	0.279
Interaction group×time	–0.42	3.50	–0.12	0.904
Intercept	45.17	4.06	11.12	0
Psychological distress (1–4)	0.18	0.15	1.20	0.230
Time	–0.03	0.04	–0.85	0.396
Interaction group×time	0.05	0.08	0.60	0.547
Intercept	2.24	0.12	17.98	0
Anxiety	0.22	0.15	1.48	0.139
Time	0.01	0.04	0.12	0.900
Interaction group×time	0.05	0.07	0.71	0.478
Intercept	1.98	0.12	16.75	0
Depression	0.16	0.17	0.94	0.346
Time	–0.06	0.05	–1.25	0.211
Interaction group×time	0.05	0.10	0.48	0.630
Intercept	2.41	0.14	17.7	0
Pain intensity in prior week (0–10)				
Current	–0.54	0.64	–0.85	0.394
Time	–0.03	0.16	–0.18	0.854
Interaction group×time	–0.12	0.31	–0.38	0.703
Intercept	5.68	0.54	10.49	0
Worst	0.17	0.42	0.39	0.693
Time	0.09	0.13	0.68	0.494
Interaction group×time	0.06	0.24	1.60	0.090
Intercept	7.50	0.34	21.84	0
Average	–0.23	0.49	–0.47	0.641
Time	0.13	0.15	0.86	0.390
Interaction group×time	0.53	0.28	1.92	0.055
Intercept	5.72	0.42	13.64	0
Least	–0.36	0.62	–0.58	0.561
Time	0.09	0.18	0.50	0.620
Interaction group×time	0.50	0.33	1.52	0.128
Intercept	3.89	0.57	6.84	0

* SE = standard error.

Linear mixed-model analysis, with time points and outcome status as fixed effects and clusters as random effects.

### Ancillary Analysis: Historical Control Group

A significantly higher proportion of the IPS group attained competitive employment during the 12-month follow-up period (n = 19, 52.8%), compared with the employment rate at the 12-month follow-up point among historical controls (n = 52, 16.5%, χ^2^ [1, n = 351] = 26.34, *P* < 0.001, OR = 5.65, 95% CI = 2.75–11.60).

## Discussion

The IPS in Pain trial investigated the effectiveness of IPS as an integrated part of interdisciplinary pain treatment for patients with chronic pain in a tertiary pain center. The main findings showed that employment rates increased throughout the follow-up period, but there was a general lack of significant differences between the groups across employment- and health-related outcomes, probably because of the low sample size.

### Competitive Employment

More than half of patients with chronic pain in the IPS group attained competitive employment during the 12-month follow-up period, which is similar to the average employment rate of 56% in trials of IPS for its original target group with SMI [30]. With employment rates increasing to about 60% after 24 months, the positive trend over time was more pronounced in the IPS group than in the TAU group. However, although the IPS group showed consistently higher employment rates, job intensity, and productivity than those of the control group, there were no statistically significant differences between the groups on any of these employment-related variables.

Interestingly, the control group in the present study showed an unexpectedly high employment rate of about 39% during 12-month follow-up, compared with 23% in previous IPS trials [30]. There are several viable explanations for this finding. First, the previous trials focused on people with SMI. Although barriers to employment can be overlapping, the employment rate of these two populations cannot be compared directly, and people with SMI face unique challenges related to employment (e.g., stigma in working life) [31]. Second, participants in the control group received interdisciplinary pain TAU, in addition to self-help advice and a resource manual focused on employment. The active nature of the control condition thereby increased the similarity between the IPS and TAU groups and is likely to have facilitated a higher employment attainment than would have been the case with a passive control condition. These notions are supported by the ancillary analysis, which showed that only 16.5% in the historical control group were employed during the 12-month follow-up period. Furthermore, it is not possible to rule out contamination bias, as both groups were treated at the same pain center. The control group also had a higher proportion of participants with higher education than did the IPS group. Although this was nonsignificant, higher education generally opens more opportunities to work.

It could also be argued that the lack of significant differences between the groups indicates no additional effect from the added component of IPS among patients with chronic pain conditions. However, the IPS group showed consistently higher rates of all employment-related variables, and the low sample size is likely to have prevented any potential effect from reaching statistical significance. This issue is further discussed in the section “Methodological Considerations.”

### Health-Related Outcomes

The IPS and TAU groups did not differ in health-related outcomes such as health-related QoL, pain-related disability, and psychological distress. The only exception was worst pain during the prior week measured 12 months after enrollment, for which the IPS group reported significantly higher pain intensity in the unadjusted analysis. Although this finding indicated that receiving follow-up with IPS directed toward employment might have exacerbated the pain, there were no significant differences in the remaining measures of pain (current, average, and least pain), and the adjusted analysis showed no overall difference between the two groups in any of the outcomes. Because of the large number of secondary outcomes, alpha inflation is a concern, suggesting that caution is needed in interpreting this single finding. Although the low sample size and relatively high attrition make it difficult to draw firm conclusions, the general lack of findings on health-related outcomes is in line with previous studies of IPS among the original target group with SMI [32].

### Methodological Considerations

The present study evaluates the promising evidence-based intervention IPS for a new and important target group by using a mix of employment- and health-related outcome measures. The preceding pilot study [16] and the randomized controlled design of the present study represent major methodological strengths. The study does, however, have some noteworthy shortcomings. Most importantly, we did not reach the predefined goal of 80 participants. The small sample size increases the risk of type II error, as the low statistical power could preclude the chance of reaching statistical significance. This limitation prevents us from making clear conclusions about effectiveness. The recruitment period could not be further prolonged, as funding was terminated after the project period. For a larger trial with a sufficient sample size of patients in this target group to be feasible, there is need for a multicenter design including several university pain clinics and including register data to reduce attrition. Efforts to achieve such a collaborative multicenter study are currently in preparation.

Because of the low sample size in the control group, data from a historical control group were collected to remediate this limitation to some extent. Meanwhile, the outcome of *current employment status at 12 months* used among patients in the historical control group is likely to be lower than the outcome of *any employment during the 12-month follow-up period* among the IPS group and is therefore not directly comparable. The results from the ancillary analysis comparing these rates should therefore be interpreted with caution.

All patients who were referred to the pain center during the recruitment period and fulfilled the inclusion criteria were eligible for participation. However, we do not have information about the exact number of patients who were assessed for eligibility, how many declined participation, or reasons for declining. This could limit the generalizability of the results. Meanwhile, a screening among all patients at the clinic in 2018 showed that 61.5% of patients who are not currently in employment would like to work. Patients without a desire to work were not eligible for the study, and the criterion of living in Oslo further excluded many patients who live outside this area.

The response rates on the primary outcome were reasonably high, ranging from 85% to 97% at the various follow-up points. The response rates on secondary outcomes were, however, relatively low, and it is possible that those lost to follow-up had different characteristics from those of respondents. Ignoring the missing data in unadjusted analyses could lead to biased and unreliable estimates. This was addressed through the use of mixed-effects regression models with maximum likelihood estimation, with adjustment for baseline characteristics and missing observations. Unadjusted and adjusted analyses yielded similar results, and results from both approaches were reported.

### Implications

The IPS in Pain trial is the first study to investigate the effect of IPS for people with chronic pain conditions. As a small study and the first of its kind, it should be regarded as a proof-of-concept trial exploring the potential of the IPS method for this new target group. The study shows that IPS can be integrated into the daily practice of interdisciplinary pain treatment and can lead to employment rates exceeding 50% in 1 year. There is a need for further research in the form of larger multicenter RCTs to shed further light on whether IPS is more effective than TAU for people with chronic pain conditions.

The IPS in Pain trial also included process measures with detailed IPS fidelity reviews, participants’ satisfaction with the intervention, and possible predictors of employment. These are beyond the scope of this article and will be reported in a future process evaluation.

## Conclusions

The IPS in Pain trial is the first study to evaluate the effect of IPS for patients with chronic pain conditions, and it explores the potential of the IPS method for this new target group. The results show that IPS can be integrated into the daily practice of interdisciplinary pain treatment, with employment rates exceeding 50% in 1 year. Participants who received IPS had consistently higher employment rates than those of the control group, but these findings did not reach statistical significance, possibly because of low sample size. Findings on health-related outcomes were also generally nonsignificant. Larger RCTs are needed in order to draw clear conclusions about effectiveness.

## Notes

Funding sources: The project was funded by the Norwegian Ministry of Health (grant number 15/5589-11) and Ministry of Labor (grant number 16/2239). The funding sources did not have any role in the study design; the collection, analysis, or interpretation of data; or the decision to submit articles for publication.

Trial registration: ClinicalTrials.gov ID: NCT02697656.
